# Genetic background influences mineral accumulation in rice straw and grains under different soil pH conditions

**DOI:** 10.1038/s41598-024-66036-7

**Published:** 2024-07-02

**Authors:** Toshio Yamamoto, Kazunari Kashihara, Tomoyuki Furuta, Qian Zhang, En Yu, Jian Feng Ma

**Affiliations:** 1 Institute of Plant Science and Resources, Okayama University, 2-20-1 Chuo, Kurashiki, Okayama 710-0046 Japan; 2 Present Address: College of Agronomy, Anhui Agriculture University, Hefei, 230036 China

**Keywords:** Plant breeding, Abiotic

## Abstract

Mineral element accumulation in plants is influenced by soil conditions and varietal factors. We investigated the dynamic accumulation of 12 elements in straw at the flowering stage and in grains at the mature stage in eight rice varieties with different genetic backgrounds (Japonica, Indica, and admixture) and flowering times (early, middle, and late) grown in soil with various pH levels. In straw, Cd, As, Mn, Zn, Ca, Mg, and Cu accumulation was influenced by both soil pH and varietal factors, whereas P, Mo, and K accumulation was influenced by pH, and Fe and Ni accumulation was affected by varietal factors. In grains, Cd, As, Mn, Cu, Ni, Mo, Ca, and Mg accumulation was influenced by both pH and varietal factors, whereas Zn, Fe, and P accumulation was affected by varietal factors, and K accumulation was not altered. Only As, Mn, Ca and Mg showed similar trends in the straw and grains, whereas the pH responses of Zn, P, K, and Ni differed between them. pH and flowering time had synergistic effects on Cd, Zn, and Mn in straw and on Cd, Ni, Mo, and Mn in grains. Soil pH is a major factor influencing mineral uptake in rice straw and grains, and genetic factors, flowering stage factors, and their interaction with soil pH contribute in a combined manner.

## Introduction

Rice (*Oryza sativa* L.) is among the most important cereal crops, as it provides most of the daily calories to more than half of the world’s population^1^, especially in Asian and African countries. The spread of modern agriculture with the increasing area of cultivated land has contributed to increases in total agricultural production globally in the past half-century. However, continuous cultivation under unfavorable field conditions may lead to soil degradation causing poor growth of crops and stagnation of crop production per unit area. In particular, 67% of cropping land worldwide is inadequate for crop growth, of which approximately half has acidic soil^2,3^ and the other half has alkaline soil^4–6^. The most deleterious factor for crops in acidic soil is thought to be toxic soluble aluminum (Al). Compared with other major crops, rice is more tolerant to soluble Al in soil^7,8^, although this tolerance is limited. Additionally, most acidic soils with soluble Al are found in tropical and subtropical regions where rice is cultivated as the staple food. In contrast, soil alkalization causes a deficiency of essential metals, such as iron (Fe), creating serious challenges to growing rice^9^. Therefore, evaluation of the influence of soil pH on rice production is important for multiple fields of agricultural research, such as breeding, cropping, physiology, and soil science^10^.

Changes in the accumulation of mineral elements are among the first responses of plants to soil pH alteration, which triggers a misbalance of homeostasis, physiological disorders caused by a deficiency or excess of specific mineral nutrients, and finally disease or growth inhibition^11^. For example, an excess of manganese (Mn) or a lack of essential elements, such as calcium (Ca), magnesium (Mg), or phosphorus (P), inhibits rice growth in acidic soil^12^. Cadmium (Cd) accumulation is also influenced by soil pH^13^. This complex phenomenon is known as the gene-by-environment interaction (G × E) of soil pH (an external environmental factor) on element uptake (a trait under genetic control), which affects both macronutrients and micronutrients.

In addition to soil pH, the accumulation of mineral elements in rice is affected by genetic and agronomical factors, such as flowering time (heading date), maturity period, and irrigation^14^. For instance, a shorter vegetative growth period with earlier flowering leads to lower Cd accumulation in grains^15^, whereas arsenic (As) exhibits an opposite pattern ^16^. These differences between early and late flowering varieties are caused by differences in the soil redox status and water content (soil moisture status) due to irrigation, drainage, and other water management determined by the flowering time^15,17^. This effect is described as the G × E of soil moisture status (an external environmental factor) on element uptake. However, there is also a gene-by-gene interaction (G × G) because the soil moisture status varies with flowering time (a trait under genetic control). Some studies have reported the pleiotropic expression of both traits, with quantitative trait loci (QTLs) for elemental uptake detected at genomic locations close to QTLs responsible for determining the flowering time^18,19^. However, it is difficult to distinguish the effect of the soil moisture status on element uptake from the effect of flowering time in the natural environment. Additionally, the effects of flowering time and soil pH on element uptake may not be independent. In the actual growing environment, these factors are intertwined, making it difficult to distinguish the effects of interactions from the effects of genetic factors.

Mineral elements are essential not only for plant growth but also for human health. Thus, understanding the regulation of their accumulation in the edible organs of crops is important. Generally, mineral elements are taken up by plant roots, and subsequently, a portion of these minerals move to the edible parts of plants, such as the grains. Therefore, it is important to understand the mechanisms of absorption during vegetative growth and translocation at the reproductive stage. Several transporter genes involved in the accumulation and translocation of nitrogen (N), P, potassium (K), Mg, sulfur (S), Mn, zinc (Zn), Fe, boron (B), and silicon (Si) have been identified in rice^20^. Recent advances in technologies for high-throughput analysis of various elements, such as inductively coupled plasma-mass spectrometry (ICP-MS), provided insight into the genetic basis of mineral element accumulation in plant organs or at specific growth stages^16,17,21–23^. These investigations revealed important clues towards understanding mineral element accumulation in plants, which involves complex genetic regulation. However, studies are needed to determine the overall dynamics of mineral elements in rice while considering the effects of environmental and genetic factors, as well as the interaction of flowering time and soil pH.

The understanding of pH-dependent variations in element absorption in paddy rice is improving with the determination of the mechanism of isolated genes. Genomic research is also underway to examine the genetic diversity of element absorption. However, to apply this knowledge in breeding and improving cultivation methods, it is necessary to comprehensively understand the dynamic accumulation of elements at different soil pH levels, taking into account the genetic background of cultivated rice plants (Japonica, Indica) and flowering time (early, medium, late), which are closely related to the environment of the growing area.

To identify the genetic factors involved in elemental absorption in response to pH changes, we grew eight rice varieties from various genetic backgrounds in fields with different soil pH levels. These eight varieties include genetic diversity between Indica and Japonica cultivated rice in East Asia. Wide genetic diversity between Indica and Japonica has been reported with respect to element absorption^24^. In addition, as these are the parental varieties of a multi-parent advanced generation inter-cross population (MAGIC) developed by Ogawa et al.^25^, the results can be effectively used for gene identification in genome-wide association studies (GWAS). We quantified 12 elements including 10 essential elements and two toxic ones in straw at the flowering stage and in harvested grains. Based on their accumulation patterns, we classified these elements into several groups.

## Materials and methods

### Plant materials

Eight varieties, Bekogonomi (BE), Akidawara (AK), Takanari (TK), Hokuriku193 (HO), Suwon258 (SU), Mizuhochikara (MI), Ruriaoba (RU), and Tachiaoba (TC), were evaluated (Table 1). HO, MI, AK, TC, TK, and BE were developed in Japan under a governmental high-yielding rice breeding program. SU is a Korean high-yielding variety and parent of MI. RU has high biomass productivity and was developed in Japan for feed production. In the context of genetic backgrounds of *O. sativa*, HO, SU, and TK belong to the Indica group; AK, TC, and BE belong to the Japonica group; and MI and RU are admixtures of Indica and Japonica, respectively. Additionally, BE and AK are early flowering; HO, TK, MI, and SU are middle flowering; and RU and TC are late flowering varieties. The plant collection and use was in accordance with all the relevant guidelines.

**Table 1:** Varieties used in the experiments.

Name of variety* (code)	Flowering time	Genetic background
Bekogonomi (BE)	Early	Japonica
Akidawara (AK)	Early	Japonica
Takanari (TK)	Middle	Indica
Hokuriku193 (HO)	Middle	Indica
Suwon258 (SU)	Middle	Indica
Mizuhochikara (MI)	Middle	Admixture
Ruriaoba (RU)	Late	Admixture
Tachiaoba (TC)	Late	Japonica

*These are listed in order from earliest to latest flowering in the experimental field.

### Field establishment and cultivation

Three separate test fields (200 m^2^ each) were constructed at the Institute of Plant Science and Resources, Okayama University (34.6°N, 133.8°E). The initial pH of the three fields measured on February 15, 2018 was 6.01 (planned for acidic), 5.89 (planned for neutral), and 5.84 (planned for alkaline), respectively. Their pH levels were subsequently adjusted to 4–5 (acidic), 6–7 (neutral), and 8–9 (alkaline) and maintained by adding soil amendments, such as hydrated lime (Lime Tec, Suzuki Industry Co., Ltd., Shizuoka, Japan) for alkaline soil or acidity regulator (Sandset, Sun Agro Co., Ltd., Tokyo, Japan) for acidic soil. To measure pH, the soil was sampled at three locations in each plot after tillage, water was added to achieve a soil:water ratio of 1:2.5, and the soil was allowed to stand for 0.5 h before measurement. For pH adjustment, the amount of acidity regulator required to lower the pH by 0.1 was calculated as 8.3 kg/a, and the amount of hydrated lime required to raise the pH by 0.1 was calculated as 3.8 kg/a based on our preliminary studies. The amounts needed to reach 4.5 (acidic), 6.5 (neutral), and 8.5 (alkaline) from the initial measurements were calculated and applied, and then promptly equalized by re-tilling. Soil pH trends for each year are shown in Supplementary Fig. S1. The physical and chemical properties of the soils in each field were analyzed at the Physical and Chemical Research Center of Vegetech Co., Ltd. (Kanagawa, Japan). Subsoil (500 g) was collected from the four corners and the center of each field, with approximately 1 cm of the soil surface removed, mixed, and air-dried for one week; 500 g of this sample was sent to the above analysis company. The results of the 2020 cropping season are shown in Supplementary Table S1.

In 2019 and 2020, seeds were sown on May 20, and seedlings were transplanted to the fields on June 20 with one plant per hill, 15 cm between hills, and 30 cm between rows. We prepared three replicated blocks in 2019 (for sampling grains) and two replicated blocks in 2020 (for sampling straw) per variety. Each block consisted of 60 plants in total arranged in three rows with 20 plants in each row. The water level was kept constant during most of the growing stages. Irrigation ended on September 1, which is the day when panicles started to emerge in almost all middle-flowering varieties. At the mature stage of each variety in 2019, eight plants in the middle row of each block were harvested and air-dried. We selected 200 mature seeds from bulked seeds derived from each block. The dehusked seeds were ground into fine powder and subjected to mineral element quantification. At the heading stage of each variety in 2020, the straws (aboveground parts) were harvested from two plants in the middle row of each block and air-dried in the net room. The plants were dried in an oven set at 70 °C for at least three days and ground into fine powder.

### Quantification of mineral elements

Two grams of the prepared powders were digested with concentrated nitric acid (60% [w/v]) at 140 °C in a heating block as described previously^26^. Concentrations of 12 elements in the digest solution were determined using ICP-MS (7700X; Agilent Technologies, Santa Clara, CA, USA).

### Statistical analysis

All analyses were conducted using R statistical software version 4.3.3 (https://www.r-project.org/)^27^. We used the standard functions implemented in the “stats” package for correlation analysis, hierarchical clustering, and two-way analysis of variance (ANOVA) with *p* < 0.05 as the cut-off for statistical significance. The data values of the quantified minerals were standardized for hierarchical clustering by z-score normalization. Statistical significances of differences among mean values were evaluated using the Tukey–Kramer multiple-comparison test.

## Results

### Stability of environmental condition and evaluation method

A comparison of days to heading of all eight varieties during the two years is shown in Supplementary Fig. S2. In 2019, the early varieties headed at 73–95 days, middle varieties at 97–110 days, and late varieties at > 113 days after seed sowing, consistent with the flowering groups listed in Table 1. Although most varieties headed slightly later in 2020 than in 2019, the overall trends were similar in both years. Additionally, the trends in culm length and panicle length were similar between years (Supplementary Fig. S3), confirming field stability. The data for grains obtained in 2021 in a different field (corresponding to the neutral condition) and data for grains obtained in 2019 in this study were similar (Supplementary Fig. S4), confirming the stability of the evaluation method. Therefore, we considered it acceptable to simultaneously evaluate mineral accumulation in grains in 2019 and straw in 2020.

### Correlation of mineral element accumulation between straw and grains

**Figure 1:**
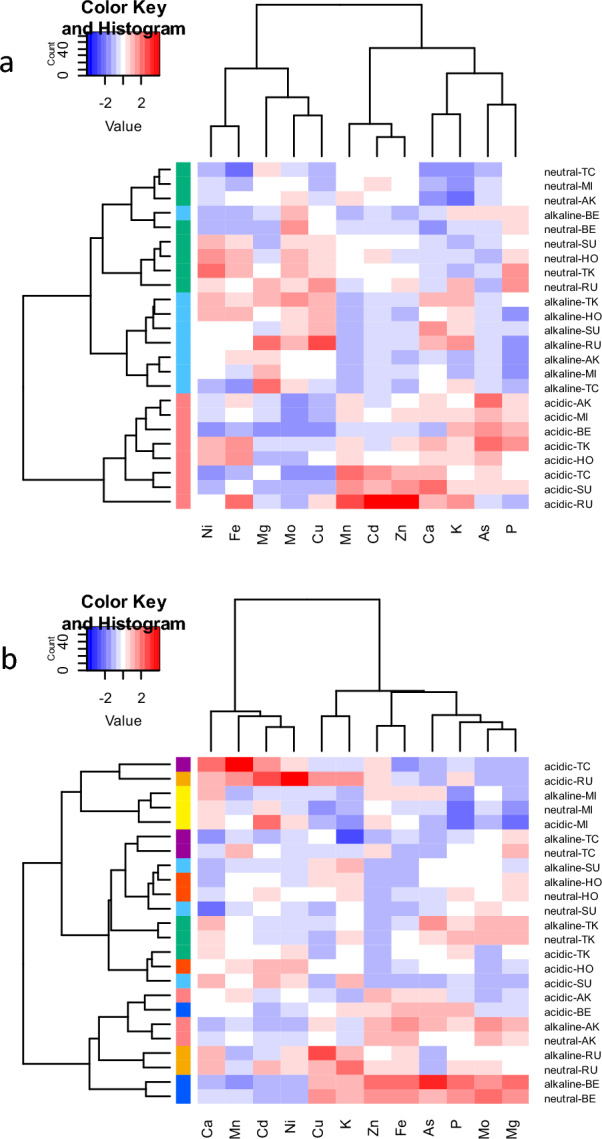
Hierarchical clustering of standardized values of concentrations of 12 elements and varieties under different soil pH levels. (**a**) Straw and (**b**) grain. Colored subcategories on the left side of each heat map represent (**a**) acidic (pink), neutral (green), and alkaline (light blue) soil, and (**b**) HO (red), MI (yellow), TK (green), BE (dark blue), SU (light blue), AK (pink), RU (orange), and TC (purple).

To assess the effects of soil pH and genetic background on mineral accumulation, we conducted hierarchical clustering analyses according to the furthest neighbor method (Fig. 1). The clustering tree for the mineral concentration data of straw showed that samples from the same soil condition were grouped (Fig. 1a), except for BE from the alkaline condition, which clustered with samples from the neutral condition. In contrast to straw, grain samples of the same variety clustered together in the tree for grains, although some varieties showed relatively higher variability under different pH conditions (Fig. 1b). These results indicate that soil pH has a stronger effect on mineral concentration in straw than did genetic factors, whereas minerals in grains are mainly genetically regulated.

We next evaluated the correlations between the accumulation patterns of the 12 elements in the samples. Positive and negative correlations were observed based on correlation coefficient thresholds of *r* > 0.5 and *r* <  − 0.5, respectively. Of the 66 combinations of the 12 elements, seven positive and five negative correlations were observed in the mineral concentrations of straw (Supplementary Fig. S5a), whereas 13 positive and five negative correlations were observed in those of grains (Supplementary Fig. S5b). Correlation in both organs was found only for the Cd-Mn pair, which was also reflected by their close positions in both clustering trees (Fig. 1). All other correlations were specific to either straw or grain. Opposite directions of correlation between straw and grain were observed in P–Mg (negative–positive) and Mo–As (negative–positive). These were also reflected by the distances between elements in each pair on the clustering trees (Fig. 1).

### Changes in mineral element accumulation at different soil pH levels

**Figure 2:**
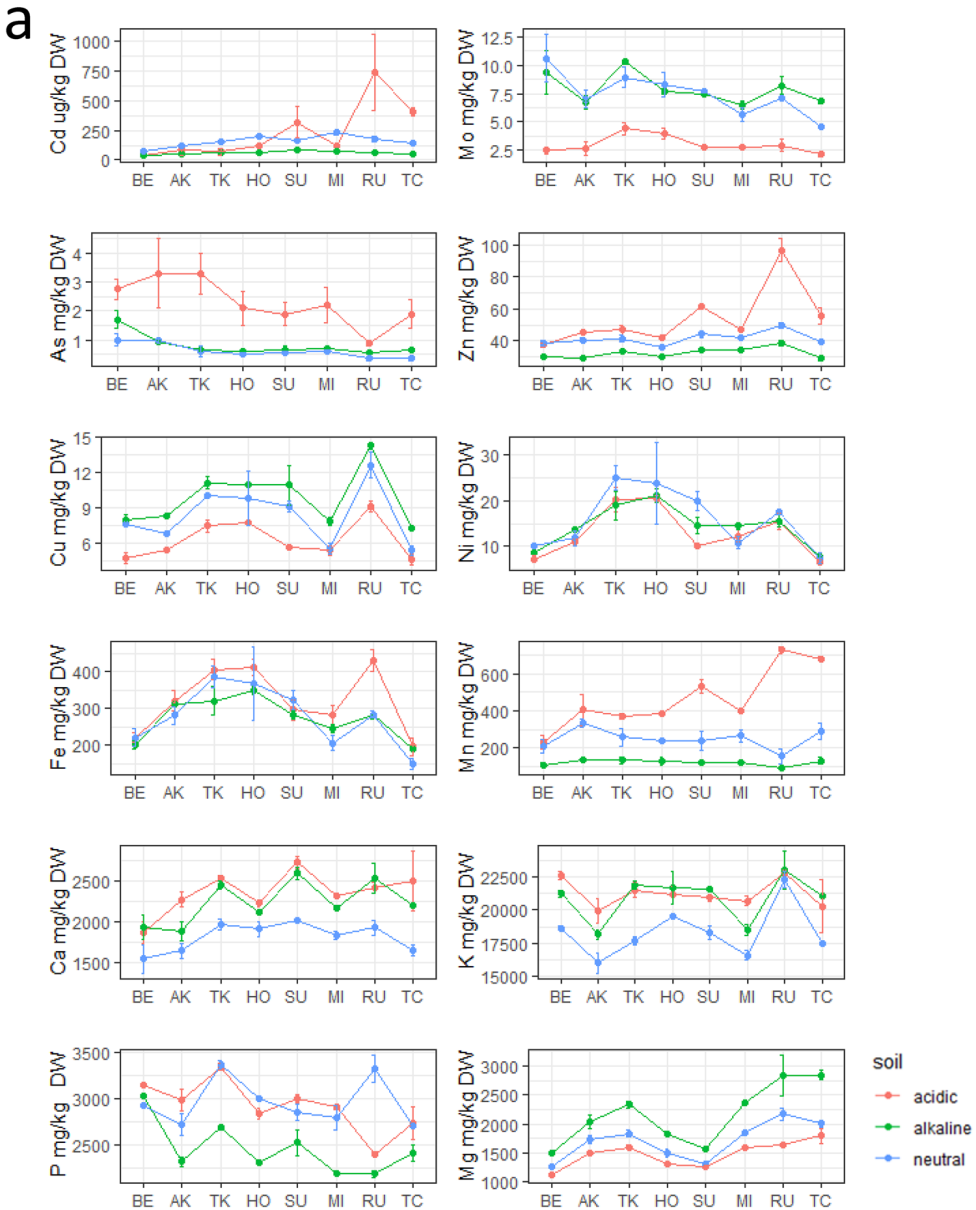

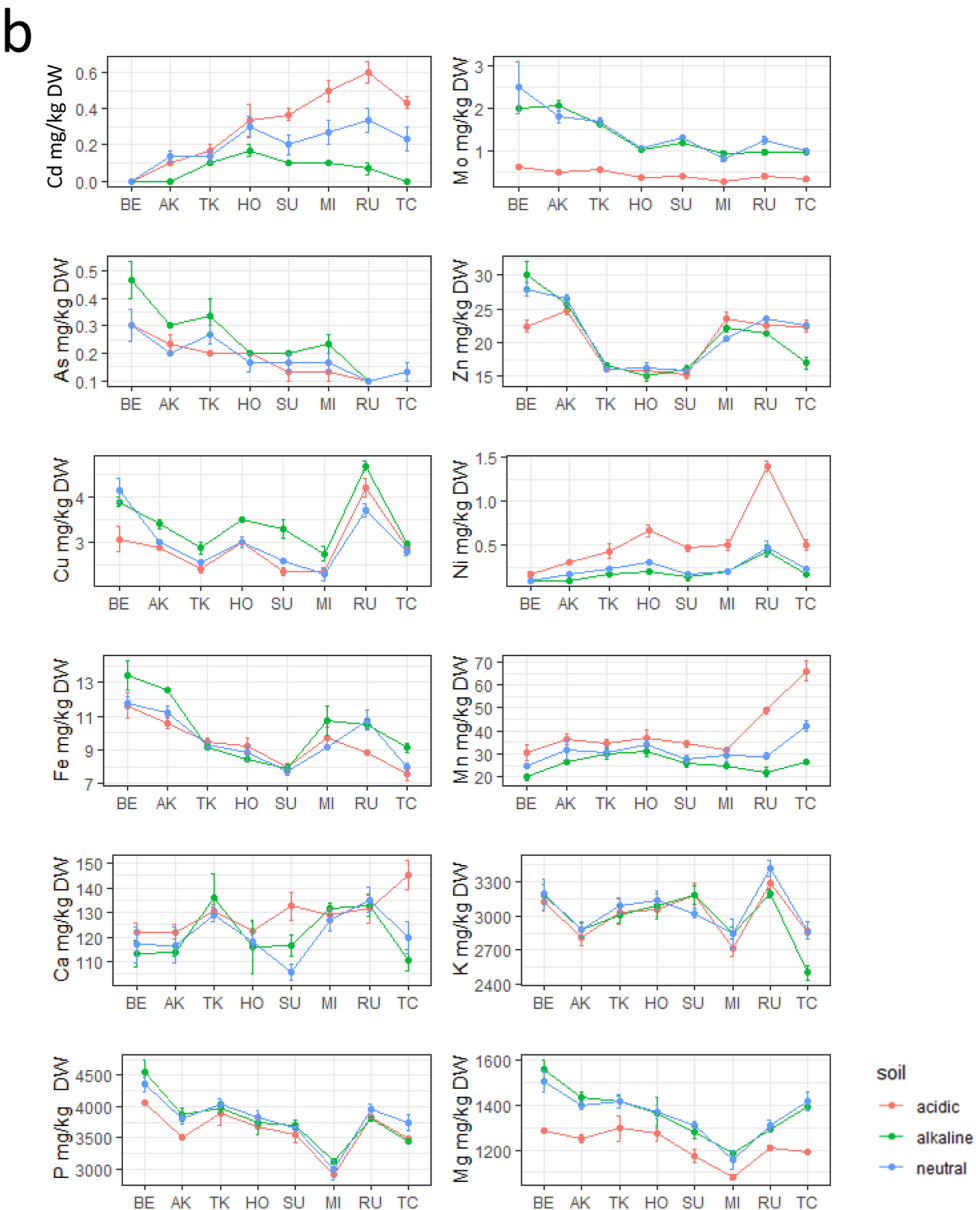
Concentrations of 12 elements in eight varieties at different soil pH levels. (**a**) Straw and (**b**) grains. Variety codes are listed in the order of days to heading to show the effect of flowering time on element accumulation.

Concentrations of the mineral elements grouped by variety and soil pH are shown in Fig. 2. Two-way ANOVA with soil pH and either flowering group (early, medium, or late) or genetic background group (Japonica, Indica, or admixture) as two factors was also performed (Supplementary Table S2). The results of the directional study for elements that were significant for soil pH are shown in Table 2. In straw, 10 elements (Cd, Mo, As, Zn, Cu, Mn, Ca, K, P, and Mg) were predicted to be sensitive or conditionally sensitive to soil pH, showing similar tendencies in all varieties (Fig. 2a, Table 2). Concentrations of Mo, Cu, and Mg tended to be low at acidic pH and high at alkaline pH, whereas those of Cd, As, Zn, Mn, and P showed the opposite trend. Ca and K concentrations were lowest at neutral pH, and Ni and Fe showed no pH dependence.

**Table 2:** Direction of change in the 12 elemental contents in acidic and alkaline soil.

Part	Element	Acidic	Alkaline	Part	Element	Acidic	Alkaline
Straw	Cd		Down	Grain	Cd	Up*	Down
	Mo	Down			Mo	Down	
	As	Up			As		Up*
	Zn	Up*	Down		Zn		
	Cu	Down	Up		Cu		Up*
	Ni				Ni	Up	
	Fe				Fe		
	Mn	Up	Down		Mn	Up	Down*
	Ca	Up	Up		Ca	Up*	
	K	Up	Up		K		
	P		Down*		P		
	Mg	Down	Up		Mg	Down	

Up/down directions were determined based on the graph in Fig. 2 for elements that were significant (pH-sensitive) in the soil based on the results of two-way ANOVA (Supplemental Table S2). Columns not marked with an asterisk (*) show this trend for all eight varieties, whereas columns marked with an asterisk (*) show this trend for five to seven varieties.

In grains, eight elements (Cd, Mo, As, Cu, Ni, Mn, Ca, and Mg) were predicted to be sensitive or conditionally sensitive to soil pH, showing similar tendencies in all varieties (Fig. 2b, Table 2). The concentrations of Mo, As, Cu, and Mg tended to be low at acidic and high at alkaline pH. The concentrations of Cd, Ni, Mn, and Ca tended to be high at acidic pH, particularly in late-flowering varieties except for Ca, whereas that of As tended to be high at alkaline pH, especially in early-flowering varieties. Four elements (Zn, Fe, K, and P) showed no pH dependence, although there were slight differences among varieties.

These results support those of clustering analyses. Interestingly, the accumulation of As in straw was highest in acidic soil, whereas in grain it was highest in alkaline soil in most varieties (Fig. 2a,b).

### Categorization of accumulation profiles of mineral elements

**Figure 3:**
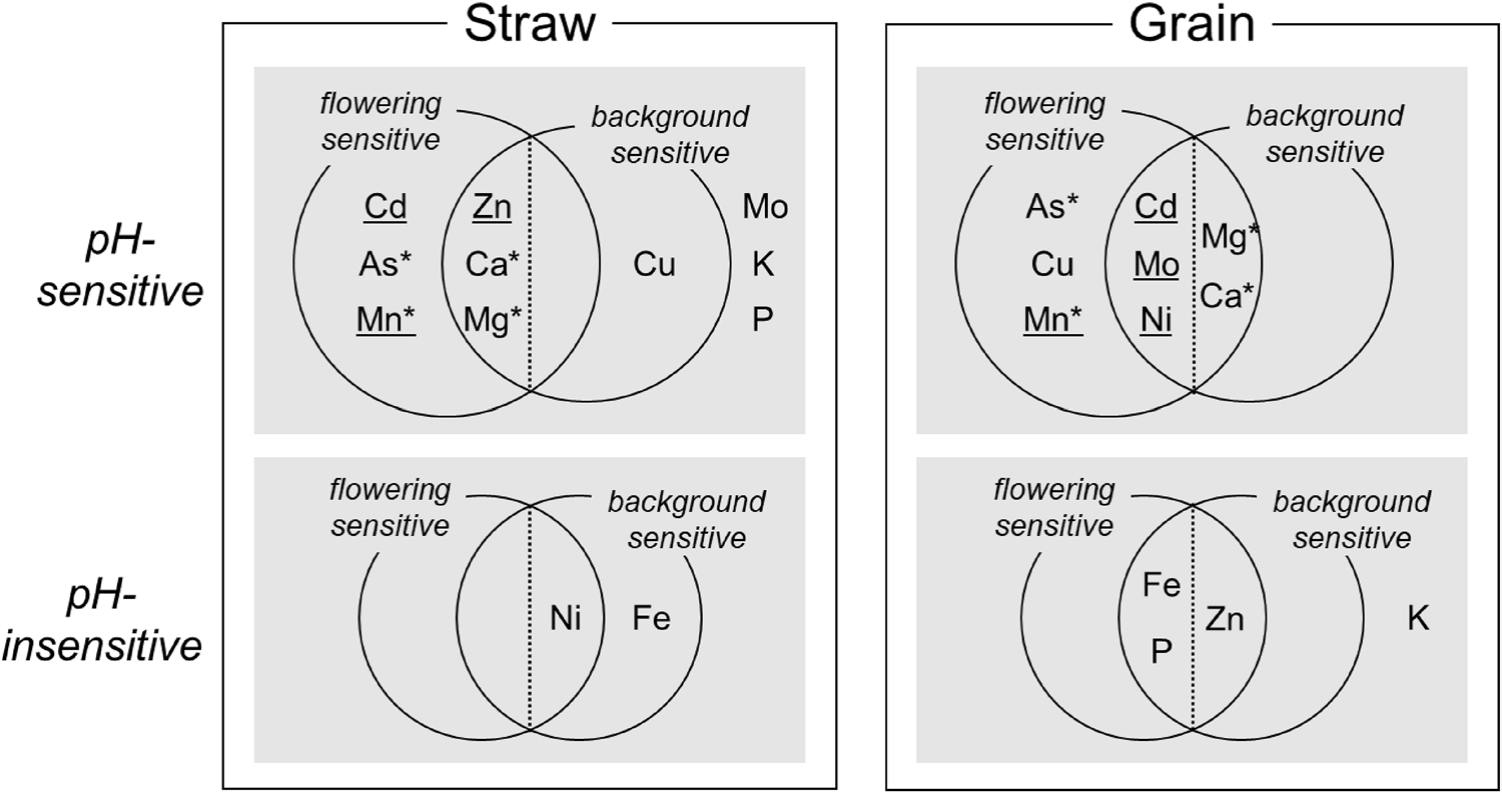
Accumulation profiles of 12 elements in straw and grains grouped by soil pH levels and flowering time and genetic background sensitivity. Underlined elements indicate the interaction between soil pH and flowering time in two-way ANOVA. Elements followed by an asterisk (*) belong to the same regions within the Venn diagram in both straw and grains.

The results of two-way ANOVA described above, with soil pH and either the flowering group or genetic background group as the two factors, were further evaluated to determine the main causal factor of the response to pH (Supplementary Table S2). In straw, the 10 elements sensitive to soil pH (Cd, As, Mo, Zn, Cu, Mn, Ca, P, K, and Mg) were divided as follows: sensitive to flowering time (Cd, As, and Mn), sensitive to genetic background (Cu), sensitive to both (Zn, Ca, and Mg), and insensitive to both (Mo, P, and K). The two elements showing insensitivity to soil pH (Fe and Ni) were divided into sensitive to genetic background (Fe) and sensitive to both flowering time and genetic background (Ni). Based on comparison of the ratio of the contribution of the four elements belonging to “sensitive to both” (Zn, Ca, Mg, and Ni) in two-way ANOVA (Supplementary Table S2), the accumulation of Zn, Ca, and Mg was more likely influenced by flowering time than by genetic background (left side of the intersection of circles in Fig. 3) and that of Ni was more likely influenced by genetic background than by flowering time (right side of the intersection of circles in Fig. 3).

In grain, the eight elements sensitive to soil pH (Cd, Mo, Cu, Ni, Mn, Ca, As, and Mg) were further divided into two groups: sensitive to flowering time (Cu, As, and Mn) and sensitive to both flowering time and genetic background (Cd, Mo, Ni, Ca, and Mg). The four elements showing insensitivity to soil pH (Zn, Fe, P, and K) were further divided into sensitive to both flowering time and genetic background (Zn, Fe, and P) and insensitive to both (K). Comparison of the ratio of contribution of the eight elements belonging to “sensitive to both” in two-way ANOVA (Supplementary Table S2) revealed that accumulation of Cd, Mo, Ni, Fe, and P was likely influenced by flowering time (left side of the intersection of circles in Fig. 3), whereas that of Mg, Zn, and Ca was likely influenced by genetic background (right side of the intersection of circles in Fig. 3).

We assigned the 12 elements in the intersections of the Venn diagrams to the side of either genetic background or flowering time (Fig. 3). The responses of several elements widely varied, as Zn, P, and K were pH-sensitive in straw and insensitive in grain, whereas Ni was pH-insensitive in straw and sensitive in grain. As, Mn, Ca, and Mg showed similar trends in both straw and grain.

### Interaction among factors

**Figure 4:**
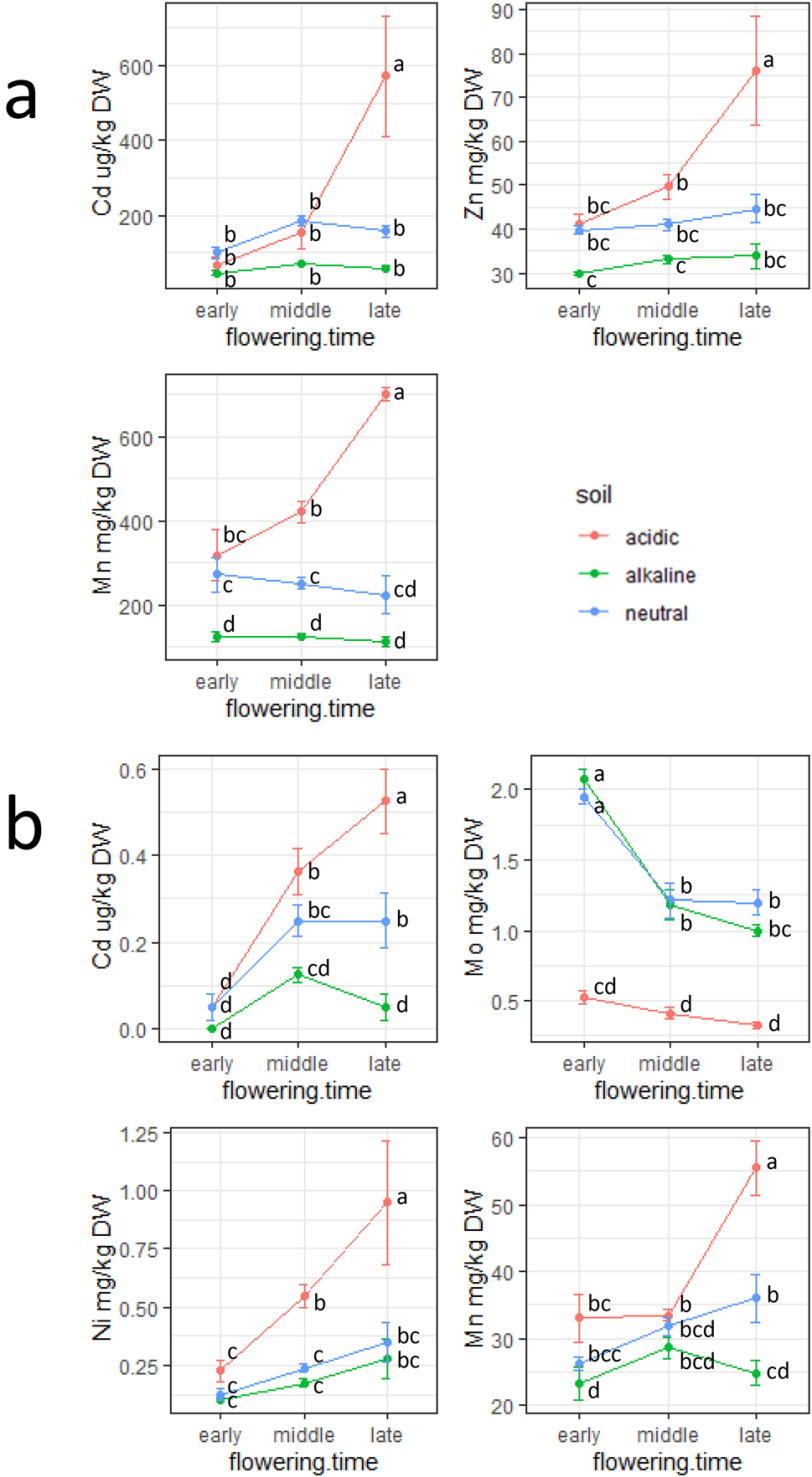
Interaction plots of accumulation of (**a**) Cd, Zn, and Mn in straw and (**b**) Cd, Mo, Ni, and Mn in grains between soil pH and flowering time. Different letter shows significant difference by Tukey–Kramer test.

Based on the results of two-way ANOVA, we detected significant interactions between soil pH and either flowering time or genetic background for Cd, Zn, and Mn in straw and for Cd, Mo, Ni, and Mn in grain (Supplementary Table S2; underlined in Fig. 3) to confirm the mode of these interactions. In straw, concentrations of Cd, Zn, and Mn were highest in late-flowering varieties in acidic soil (Fig. 4a). In grain, concentrations of Cd, Ni, and Mn were highest in late-flowering varieties in acidic soil, whereas that of Mo was highest in early-flowering varieties in alkaline and neutral soils (Fig. 4b).

## Discussion

Several comprehensive GWAS of mineral element accumulation in a diverse rice collection under different conditions have been reported. For example, Yang et al.^16^ evaluated 17 elements in grains and shoots of 529 accessions under low and high fertilizer (N and P) conditions. Liu et al.^17^ evaluated 16 elements in grains of 191 accessions under anaerobic and aerobic conditions. Tan et al.^22^ evaluated 16 elements in grains of 575 accessions in eight environments including different sites and years. As the latest example, Cobb et al.^23^ evaluated 20 elements in the roots and shoots of seedlings of 373 diverse rice lines. These studies illustrated the importance of understanding the genetic determinants of mineral element dynamics in rice under different environmental conditions. In this study, we investigated the concentrations of 12 elements in straw and grain under different soil pH levels.

### Pairwise correlations between elements and comparison between straw and grains

The overall accumulation profiles (Fig. 1) and correlation patterns (Supplementary Fig. S5) of the 12 elements were inconsistent between straw and grain, agreeing with results reported by Yang et al.^16^. Only the Cd–Mn pair showed a strong positive correlation in both straw and grain, likely because of their dependence on the same transporters^26,28^. In contrast, strong negative correlations were observed for P–Mg and Mo–As in straw, whereas strong positive correlations were observed in grain. Taken together, these data suggest that uptake from the soil through the roots and distribution within the plant body are controlled by different mechanisms. The two major breeding objectives in genetic improvement of mineral element accumulation in rice are the utilization of culms or leaves (e.g., for phytoremediation or livestock feeding) and the utilization of organs edible by humans (e.g., as nutrient supplements or to treat health problems). Because of differences in how mineral elements are allocated to straw and grain, these two objectives should be pursued using two different breeding strategies.

### Mineral element accumulation is strongly affected by soil pH

In straw, despite some differences among varieties, the concentrations of As, Mn, and Zn increased and those of Mo, Cu, and Mg decreased in acidic soil, whereas those of Cu and Mg increased and those of Cd, Zn, Mn, and P decreased in alkaline soil (Fig. 2a, Table 2). Mo levels are low in plants grown in acidic soil because this element is strongly adsorbed by reactive iron oxides and hydroxides^29,30^. Because Mn is highly soluble in acidic soil, its concentrations tend to be high in rice and other plant species^31,32^. In alkaline soil, P^33^, Zn, and Mn^34^ are present in forms unavailable to plants, resulting in their low uptake. These trends were confirmed in our study. However, the trends for some elements contrasted those reported previously. For example, concentrations of available Ca, K, and P were reported to be low in acidic soil^35^. The higher concentrations of these elements in acidic soil in our study may be associated with differences in physicochemical factors among fields (Supplementary Table S1).

The dependence of mineral element accumulation on soil pH has not been studied as extensively in grain compared with straw, except for Cd^13^. We observed interesting trends for Cd and As (Fig. 2b, Table 2), which are important toxic elements that cause human health problems. Their uptake in rice straw and grain in relation to the heading date has been widely examined^14,15,19^. Because Cd uptake is lowered by irrigation and is promoted under drainage because of differences in Cd availability in soil, water availability after flowering may be a major determinant of its accumulation. As uptake is enhanced under irrigation because of the reduction of As from As(V) to As(III). Therefore, among the several rice varieties grown under the same cropping management in the same field in this study, early-heading varieties tend to have high As and low Cd contents, whereas late-heading varieties tend to have low As and high Cd contents (Fig. 2b, Table 2). However, the Cd content increased in late-heading varieties under acidic conditions in both straw and grain, whereas the content of As in early-heading varieties increased under acidic conditions in straw but increased under alkaline conditions in grains. These results suggest that differences in soil pH conditions alter the accumulation pattern of these minerals, which is defined by differences in flowering time. Several genes involving the accumulation of these mineral elements have been isolated. *HMA3*^36,37^ and *Nramp5*^26,28,38,39^ encode Cd transporters, whereas *OsABCC1*^40^, *OsPT8*^41^, *OsNIP1;1*, and *OsNIP3;3*^42^ encode As transporters. Whether these genes are involved in the process through which variability in soil pH causes differences in mineral absorption by straw and grain must be further examined to provide information for reducing the content of both heavy metals in rice tissues.

### Categorization of mineral elements based on uptake response

We classified the 12 elements into four types: (1) pH-sensitive in straw and grains (Cd, As, Mn, Mg, Cu, Mo, and Ca), (2) sensitive in straw but insensitive in grains (Zn, K, and P), (3) insensitive in straw but sensitive in grains (Ni), and (4) insensitive in straw and grains (Fe). We detected 10 pH-sensitive elements in straw and eight in grains, whereas we found only two pH-insensitive elements in straw and four in grains. Soil pH influenced the overall profile of mineral element accumulation. The relationships between soil pH and increases or decreases in the accumulation of type 1 elements were the same for all elements. This result implies relatively low genetic variation in the system for the translocation and distribution of these elements in the tested varieties of cultivated rice. For type 2 elements, accumulation was influenced by soil pH. A neutralization or feedback system may work during translocation and distribution to grain, which could restore stability. Although soil pH did not influence the uptake of type 3 elements, the translocation and distribution to grain may be influenced by soil pH and therefore is elevated in acidic soil. Finally, we found that the translocation and distribution of type 4 elements is influenced by genetic background rather than by soil pH.

In straw, the accumulation of 10 elements was influenced by soil pH, the accumulation of As, Cd, and Mn was simultaneously influenced by flowering time, the accumulation of Cu was simultaneously influenced by genetic background, and the accumulation of Zn, Mg, and Ca was simultaneously influenced by both flowering time and genetic background (Fig. 3). Duan et al.^14^ discussed the positive and negative correlations of grain mineral elements (Cd and As) uptake and days to heading. One of the likely explanations of this flowering time sensitivity is influence of irrigation during growth ^15^, because we drained the water after heading of HO (middle-flowering). Accumulation of Mo, P, and K was affected only by soil pH and thus was not controlled by genetic variation. Variations in Ni and Fe, which were not influenced by soil pH, were likely regulated by genetic factors other than flowering time (Fig. 3). Japonica varieties (BE, AK, and TC) tended to have lower contents of Ni and Fe than did Indica varieties (TK, HO, and SU) (Fig. 2a). Ikehashi^11^ reviewed multiple genetic variations in rice that affect its responses to soil Fe deficiency and excess.

In grains, the accumulation of Cu, Mn, As, Cd, Mo, Ni, Ca, and Mg was influenced by soil pH and simultaneously by flowering time but not by genetic background alone (Figs. 2b and 3). Accumulation of Zn, Fe, P, and K in grain was stable regardless of the soil pH levels. The contents of Fe and P may have been influenced by flowering time, whereas that of Zn may have been influenced by genetic background based on its specific profiles in Indica varieties (TK, HO, and SU) (Figs. 2b and 3). These results suggest that translocation and distribution of these elements into grain are controlled directly by genetic factors such as different transporter genes. QTLs involved in Zn and Fe accumulation in grain were detected in a population of rice cultivars of different genetic backgrounds^43,44^. Moreover, only Mn, As, Mg, and Ca were in the same regions in the Venn diagram in straw and grain (asterisks in Fig. 3). To control the concentrations of these four elements, it is necessary to understand the genetic basis of their uptake from the root to the plant body.

We observed interactions of mineral element accumulation with soil pH and flowering time (Fig. 4). A typical synergistic effect was an increase in concentrations by a combination of late-flowering in acidic soil (Cd, Zn, and Mn in straw and Cd, Ni, and Mn in grains). In our experimental design, the late-flowering varieties were exposed to a drained environment which could have caused simultaneous oxidation and acidification, affecting the uptake of Cd and Mn. This can be supported since these two elements show similar profiles (Supplementary Fig. S5). As noted by Dong et al.^45^, changes in the expression of transporter genes under different soil pH and moisture conditions as well as aboveground microenvironments should be evaluated. In contrast, Mo showed a unique pattern in grains, which was higher under non-acidic conditions in early-flowering varieties than in other varieties. Mo accumulation is negatively correlated with flowering time^46^; however, we hypothesized that the negative effect of acidic soil overwhelms the advantage of early flowering because Mo availability is very low in acid soils^29^. Because genetic control of both flowering time and transporter gene expression is variable and complicated, we were unable to further examine this effect; nonetheless, the gene–environment interaction is indispensable in the regulation of mineral element uptake. In a pioneering study, Young et al.^47^ clarified the comprehensive relationship of the transcriptome and mineral elemental accumulation between the rhizosphere and plant at different soil pH levels.

### Toward the application of our findings to rice breeding and cultivation

There were some limitations to this study. First, with respect to differences in elemental absorption among varieties, the relationship between the results obtained for the eight varieties (mainly Japonica and Indica varieties developed in Japan) and the generally recognized morphological, anatomical, and physiological characteristics of Japonica and Indica varieties^48^ is not clear. However, as these eight varieties are the founders of a MAGIC population^25^, a similar evaluation in this population would identify genes involved in pH condition-specific mineral element accumulation, as well as the relationship between genes involved and typical characteristics.

Second, although the test soils used were carefully adjusted for pH, it is unclear whether the remaining characteristics mimic actual inferior environments, which may affect the generalizability of the results. For example, environmental factors such as electrical conductivity (EC) and physical properties may also affect element absorption, and there are synergistic and antagonistic effects related to the solubility of elements in the soil. The present results may be an example of detection independent of the status of such factors, and the data of soil analysis such as Supplementary Table S1 should always be considered in conjunction with them. Mineral elements are important targets for regulating the contents of nutrients and other ingredients of plant-based products and for crop growth. Our results on the overall responses of mineral elements to various soil conditions can be used to control the levels of these elements in rice breeding and cultivation practices.

## Conclusions

We investigated the concentrations of 12 mineral elements in straw at the flowering stage and in the grain of eight rice varieties with different genetic backgrounds and flowering times grown under different field soil pH levels. The trends in the accumulation profiles of most elements differed between straw and grains. Based on the categorization of elements according to their accumulation patterns, we suggest that gene–by-environment interactions are indispensable for the regulation of mineral element accumulation. Our findings will contribute to rice cultivation in different soil environments and to improving mineral element accumulation in sustainable rice breeding.

### Supplementary Information

Supplementary Figures.

Supplementary Tables.

## Data Availability

The data sets generated during and/or analyzed during the current study are available from the corresponding authors on reasonable request.
