# Supplementary material for: Genetic background influences mineral accumulation in rice straw and grains under different soil pH conditions
Source: Sci Rep. 2024 Jul 2;14:15139. doi: 10.1038/s41598-024-66036-7 (PMC11220084; doi:10.1038/s41598-024-66036-7)
Supplement: Supplementary file 1 — Supplementary Figures. [file 41598_2024_66036_MOESM1_ESM.pdf]

Supplemental Information file

Genetic background influences mineral accumulation in rice straw and grains under different soil pH conditions

Toshio Yamamoto [1,*], Kazunari Kashihara [1], Tomoyuki Furuta [1], Qian Zhang [1], En Yu [1,2], Jian Feng Ma [1]

1 Institute of Plant Science and Resources, Okayama University, 2-20-1 Chuo, Kurashiki, Okayama 710-0046, Japan
2 College of Agronomy, Anhui Agriculture University, Hefei 230036, China

*Corresponding author:
Toshio Yamamoto; yamamo101040@okayama-u.ac.jp; Tel.: +81-86-434-1205

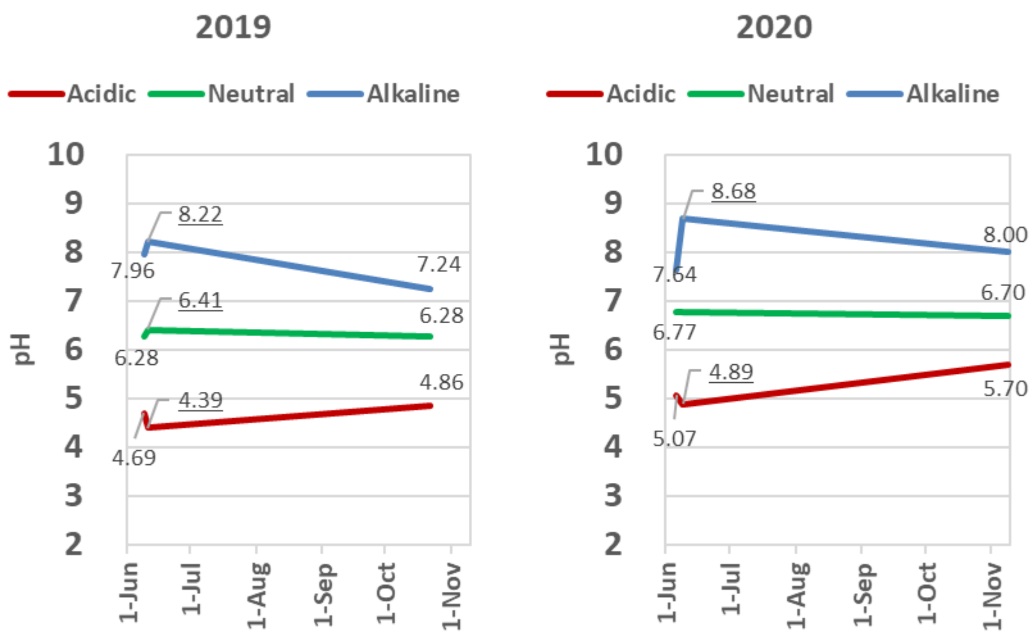

Supplementary Fig. S1. Changes in pH before and after the experiment
In 2019, soil pH was measured on June 9 after tillage; based on the results, the pH was adjusted on June 11, 2019. Acidity regulator (50 kg) was applied to the acid field, 10 kg of hydrated lime was applied to the neutral field, and 20 kg of hydrated lime was applied to the alkaline field. Soil pH was measured again on October 22 after tilling at the end of the growing season. In 2020, the pH was measured on June 6; based on the results, the pH was adjusted on June 9, 2020. In the acid field, 30 kg of acidity regulator was applied and 80 kg of hydrated lime was applied in the alkaline field. Soil pH was measured again on November 9 after tilling at the end of the growing season. Predicted pH values after adjustment are underlined.

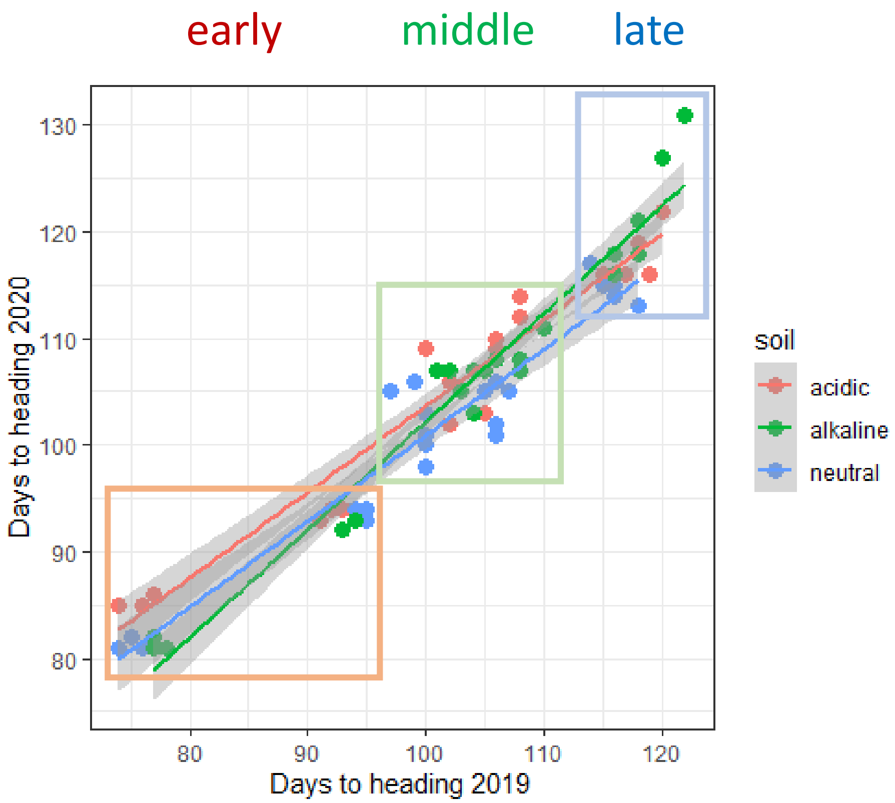

Supplementary Fig. S2. Comparison of days to heading at different field soil pH levels in 2019 and 2020. Each dot represents one replication. The "early," "middle," and "late" categories refer to flowering time and correspond to those in Table 1.

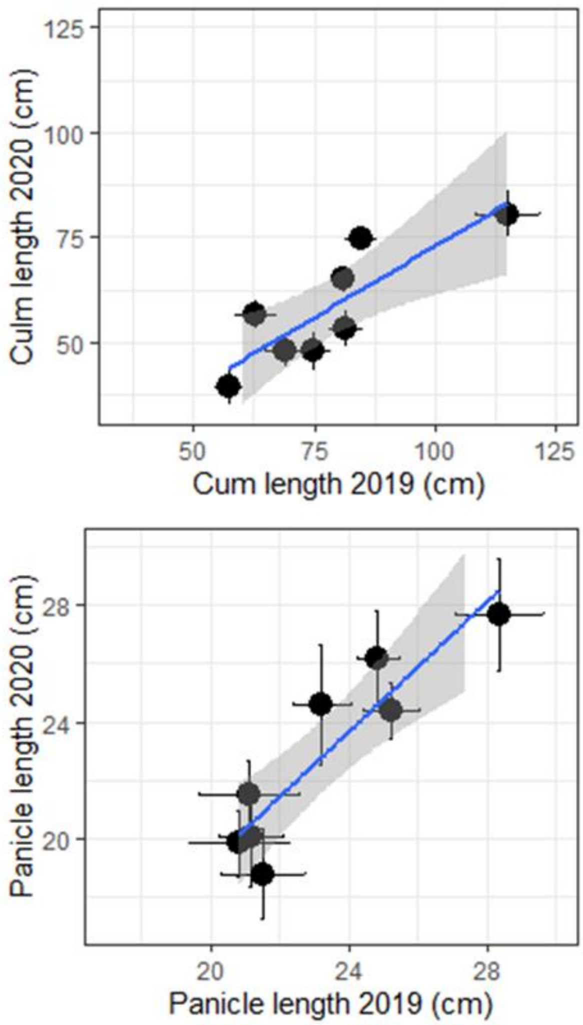

Supplementary Fig. S3. Comparison of days to heading, culm length and panicle length of eight varieties in 2019 and 2020. Each dot represents mean value and sd.

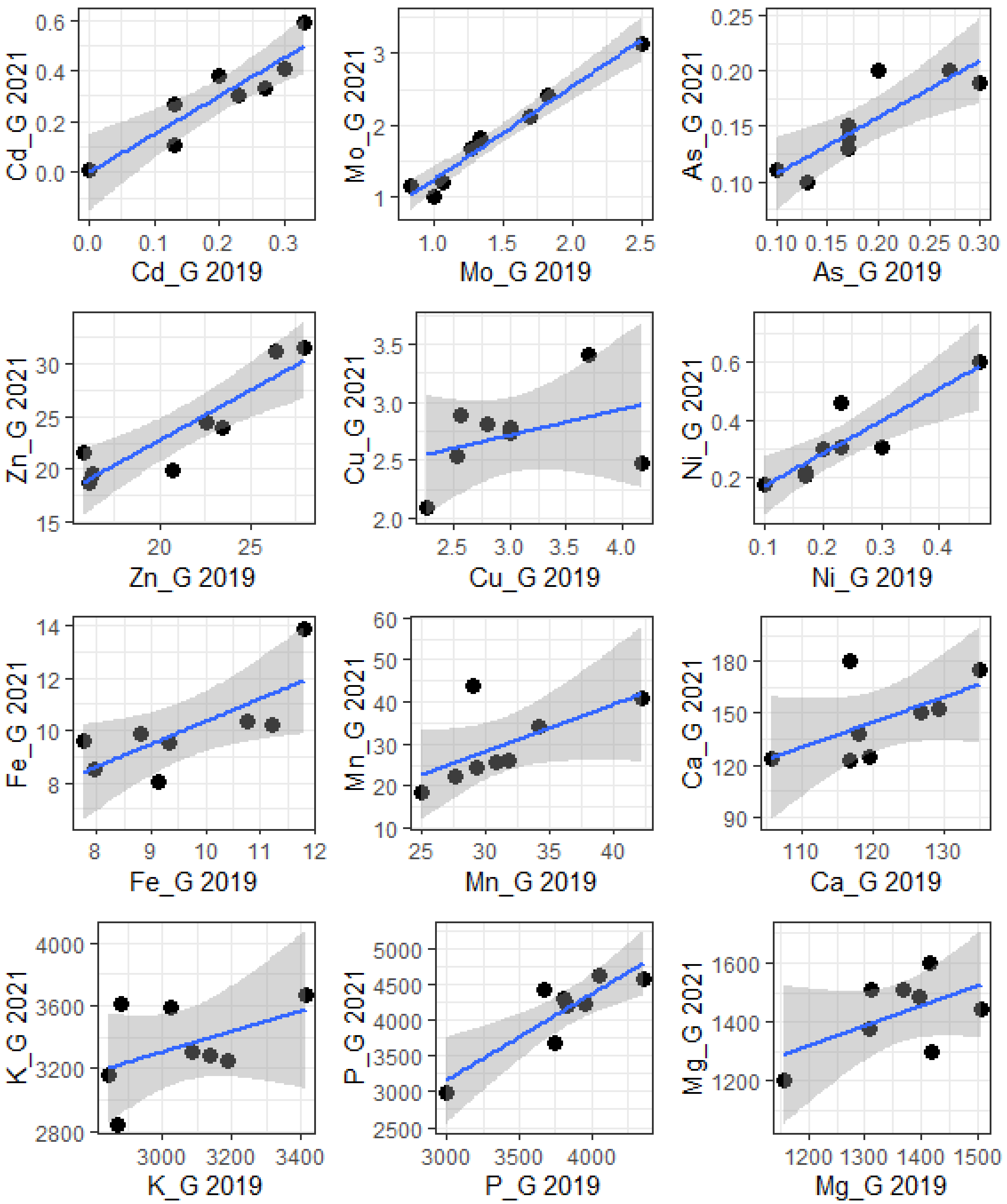

Supplementary Fig. S4. Comparison of results from two years (2019 and 2021) for the content of 12 elements of grain in the eight rice verities.

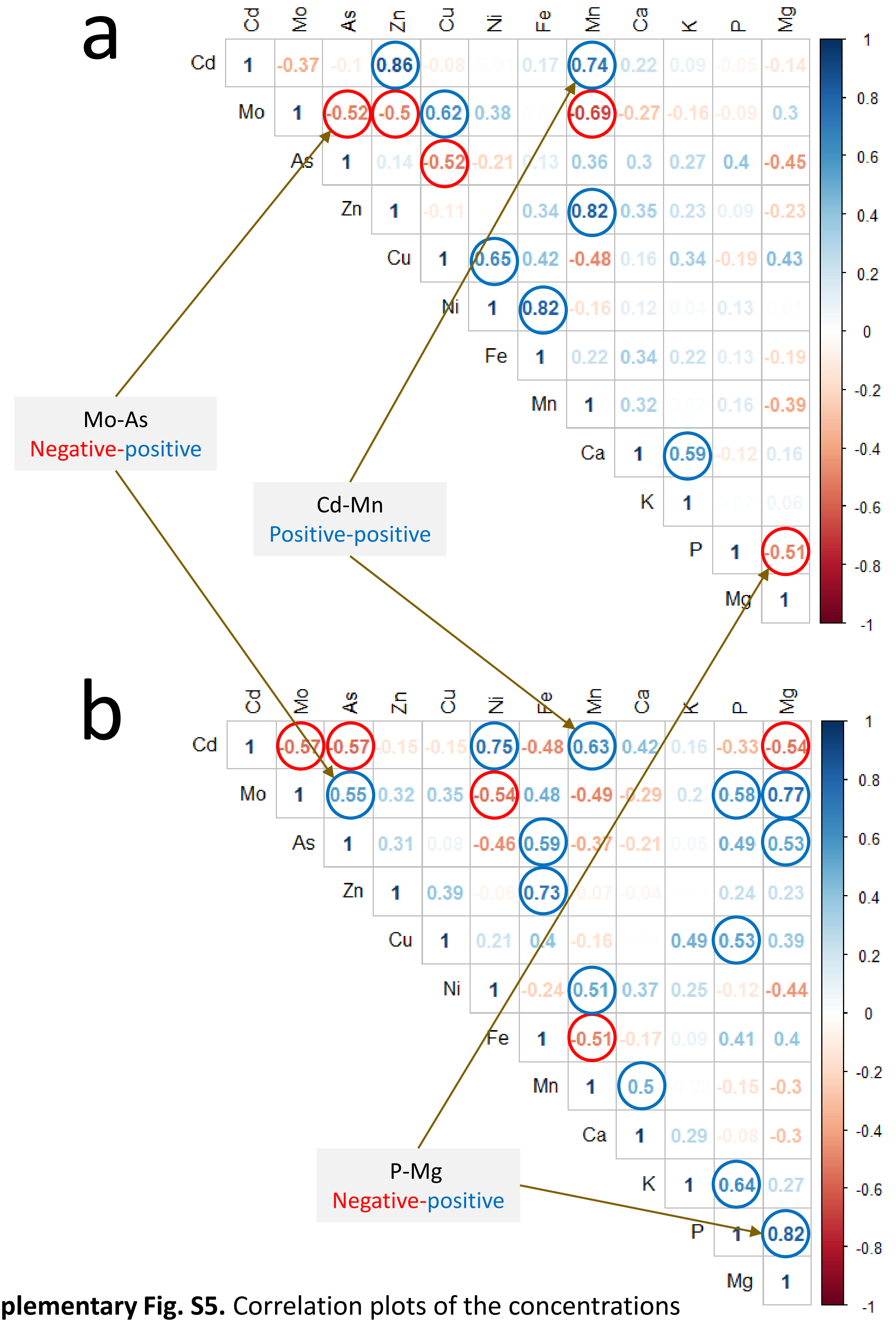

Supplementary Fig. S5. Correlation plots of the concentrations of 12 elements. (**a**) Straw and (**b**) grains. Circled correlation coefficients represent $|r| > 0.5$.
